# Comparative Analysis of Chloroplast Genomes of Four Medicinal Capparaceae Species: Genome Structures, Phylogenetic Relationships and Adaptive Evolution

**DOI:** 10.3390/plants10061229

**Published:** 2021-06-17

**Authors:** Dhafer A. Alzahrani, Enas J. Albokhari, Samaila S. Yaradua, Abidina Abba

**Affiliations:** 1 Department of Biological Sciences, Faculty of Sciences, King Abdulaziz University, P.O. Box 80203, Jeddah 21589, Saudi Arabia; dryaradua@gmail.com (S.S.Y.); abidin2007@gmail.com (A.A.); 2 Department of Biological Sciences, Faculty of Applied Sciences, Umm Al-Qura University, Makkah 24351, Saudi Arabia

**Keywords:** Capparaceae, chloroplast genome, *Cadaba*, *Maerua*, phylogenetic relationships

## Abstract

This study presents for the first time the complete chloroplast genomes of four medicinal species in the Capparaceae family belonging to two different genera, *Cadaba* and *Maerua* (i.e., *C. farinosa, C. glandulosa, M. crassifolia* and *M. oblongifolia*), to investigate their evolutionary process and to infer their phylogenetic positions. The four species are considered important medicinal plants, and are used in the treatment of many diseases. In the genus *Cadaba*, the chloroplast genome ranges from 156,481 bp to 156,560 bp, while that of *Maerua* ranges from 155,685 bp to 155,436 bp. The chloroplast genome of *C. farinosa, M. crassifolia* and *M. oblongifolia* contains 138 genes, while that of *C. glandulosa* contains 137 genes, comprising 81 protein-coding genes, 31 tRNA genes and 4 rRNA genes. Out of the total genes, 116–117 are unique, while the remaining 19 are replicated in inverted repeat regions. The *psbG* gene, which encodes for subunit K of NADH dehydrogenase, is absent in *C. glandulosa*. A total of 249 microsatellites were found in the chloroplast genome of *C. farinosa*, 251 in *C. glandulosa*, 227 in *M. crassifolia* and 233 in *M. oblongifolia*, the majority of which are mononucleotides A/T found in the intergenic spacer. Comparative analysis revealed variable hotspot regions (*atpF*, *rpoC2*, *rps19* and *ycf1*), which can be used as molecular markers for species authentication and as regions for inferring phylogenetic relationships among them, as well as for evolutionary studies. The monophyly of Capparaceae and other families under Brassicales, as well as the phylogenetic positions of the studied species, are highly supported by all the relationships in the phylogenetic tree. The cp genomes reported in this study will provide resources for studying the genetic diversity of Capparaceae, as well as resolving phylogenetic relationships within the family.

## 1. Introduction

The family Capparaceae, whose members are distributed in both arid and semi-arid areas, has about 470 morphologically diverse species in ca. 17 genera, which include *Cadaba* and *Maerua* [1,2,3]. Members of the family possess highly essential compounds used in folk medicine that are extracted from them [4]. The four species in question are considered important medicinal plants and are used in the treatment of many diseases. Most *Cadaba* species contain important compounds, such as alkaloids, sesquiterpene lactones and cadabicine. *Cadaba farinosa* and *Cadaba glandulosa* are used as purgative, anthelmintic, antisyphilitic, emmenagogue, aperient, antiscorbutic, and antiphlogistic substances; for liver damage and cancer, dysentery, fever and body pain; in therapy as a hepatoprotective and hypoglycemic [5,6]. *Maerua crassifolia* and *Maerua oblongifolia* species are used in the treatment of fever, stomach troubles, skin infections, diabetes mellitus, epilepsy and abdominal colic; they demonstrate antimicrobial and antioxidant properties and are used for hypocholesterolemia, wound-healing, intestinal disorders like abdominal cramps and hookworms, anthrax, severe mumps, tetanus and eye disease [5,7,8,9,10,11].

According to the taxonomic status of Capparaceae, Capparideae was placed under the cohort Parietales [12]. Later, Capparidaceae and Cruciferae were placed under suborder Capparidineae, order Rhoedales [13], and Capparidaceae was classified under Capparidales [14,15]. After some decades, Capparaceae was placed under Capparales [1,16], and finally under order Brassicales [17,18,19,20]. Previous studies, with the exception of Hutchinson [14], reported that Brassicaceae and Capparaceae are sister taxa [1,21,22,23,24,25,26,27,28,29,30]. The two families (Brassicaceae and Capparaceae) are considered as one family—Brassicaceae sensu lato (s.l.)—by some authors [17,18,31,32]. Phylogenetic relationship studies using genes from chloroplast and nuclear genomes [29,33] confirmed the monophyly of Brassicaceae and Capparaceae. Within Capparaceae, there are two subfamilies, Cleomoideae and Capparoideae; these subfamilies are elevated to family by some studies of Brassicales [14,34]. Currently, as adopted by the Angiosperm Phylogeny Group [19,20], Cleomaceae, Capparaceae and Brassicaceae are considered as a single family.

There has been some shifting of a few genera between the families Brassicaceae and Capparaceae, such as two genera, *Dipterygium* and *Puccionia*, previously belonging to Brassicaceae [14] being moved to Capparaceae under the subfamily Dipterigioideae, based on the presence of methyl-glucosinolate [35,36]. The genus *Stixis* L. was removed from the Capparaceae family and represents as a new family, which is called Stixaceae Doweld (including the genus *Forchhammeria* Lieb.), yet, it is still considered under Brassicaceae sensu lato, excluding *Forchhammeria,* as it is more closely related to Resedaceae than Brassicaceae [37].

Genetic information is a reliable means of understanding evolutionary relationships among species in various taxonomic hierarchies. The genetic information in the chloroplast genome contains sufficient information for comparison analysis and studies of species diversification, due to the presence of functional genes that have a vital role in plant cells [38]. The chloroplast organelle functions in carbon fixation and photosynthesis in plants [39]. The chloroplast genome is more conserved than other genomes found in plants. Generally, the chloroplast genome is circular, double-stranded and has a quadripartite structure, including a large single copy (LSC), as well as a small single copy (SSC) and a pair of repeats (IRa and IRb) [40]. The chloroplast genome is uniparentally inherited, and this characteristic makes it highly conserved in structure and gene content [41,42]. However, different kinds of mutations do occur [43], which consequently lead to sequence divergence among species and could be used to study evolutionary relationships in plants [44]. Despite the importance of the plastome in modern taxonomy, chloroplast genomes of only three species in the whole Capparaceae family, including three varieties, have been reported: *Capparis spinosa* [45], *Capparis spinosa* var. *spinosa*, *Capparis spinosa* var. *herbacea*, *Capparis spinosa* var. *ovata* [46] and *Capparis decidua*.

This study obtained the first complete chloroplast genome of the genus *Cadaba* (*Cadaba farinosa* and *Cadaba glandulosa*) and genus *Maerua* (*Maerua crassifolia* and *Maerua oblongifolia*) using Illumina sequencing technology. This study also analyzed and compared the features of the cp genomes to provide resources of genetic data for the four species. We reconstructed the phylogenetic relationship between Capparaceae, Cleomaceae and Brassicaceae to infer the phylogenetic positions of the species within the families.

## 2. Results

### 2.1. Characteristics of Four Chloroplast Genomes

**Figure 1:**
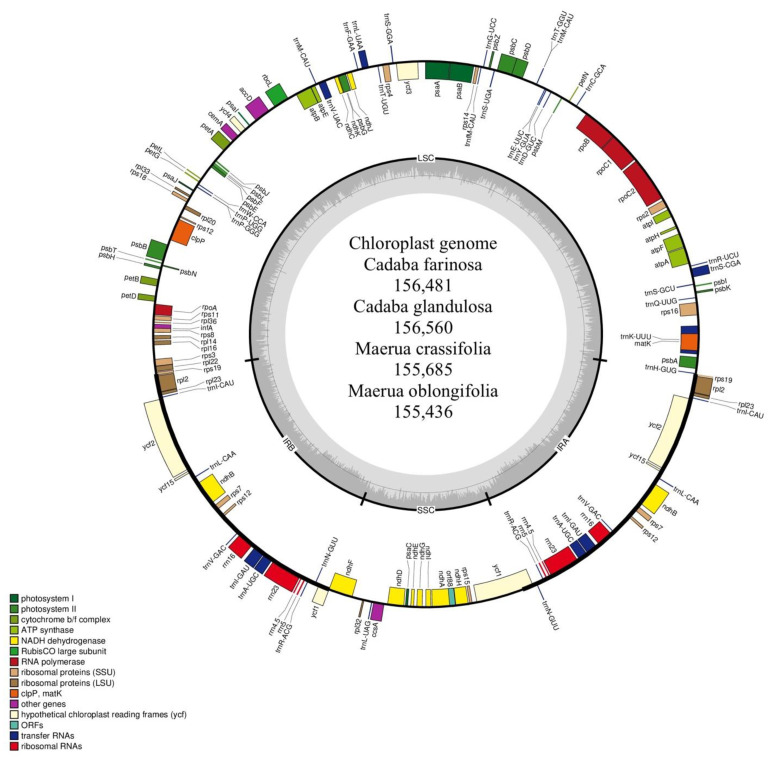
Chloroplast genome maps of the four Capparaceae species. Genes drawn inside the circle are transcribed clockwise, while those outside the circle are transcribed counter-clockwise. The inner dark gray circle corresponds to GC content and the inner light gray circle corresponds to the AT content. Different colors are used as a representation of distinctive genes within separate functional groups.

Previous studies have shown that the plastomes of flowering plants are greatly conserved in structural organization and gene content, but contraction and expansion do occur [47,48]. Each complete chloroplast genome of *C. farinosa, C. glandulosa, M. crassifolia* and *M. oblongifolia* has a circular and quadripartite structure. The genome of *C. farinosa, C. glandulosa, M. crassifolia* and *M. oblongifolia* ranged from 156,560 bp (*C. glandulosa*) to 155,436 bp (*M. oblongifolia*); the coding region ranged from 78,080 bp (*C. farinosa*) to 76,614 bp (*C. glandulosa*), corresponding to 49.89% and 48.93% of the total genome length. The LSC regions ranged from 85,681 bp (*C. glandulosa*) to 84,153 bp (*M. oblongifolia*) in size, whereas the SSC ranged from 18,481 bp (*M. oblongifolia*) to 18,031 bp (*C. glandulosa*); the pair of inverted repeats are separated by the small single copy region and ranged from 26,430 bp (*C. farinosa*) to 26,294 bp (*M. crassifolia*) (Table 1 and Figure 1). These four Capparaceae chloroplast genome sequences were deposited in GenBank (accession numbers: *C. farinosa*, MN603027; *C. glandulosa*, MN603028; *M. crassifolia*, MN603029 and *M. oblongifolia*, MN603030). 

In the four species, the plastomes are well conserved in gene order and number of genes, with slight variation in the presence of the *psbG* gene, which is absent in *C. glandulosa*. The result of the gene annotation revealed a total of 137 in *C. glandulosa* and 138 genes in *C. farinosa, M. crassifolia* and *M. oblongifolia*, among which 116–117 are situated in the SSC and the LSC copy regions, and 19 genes are located in the IRa and IRb. The plastome contained 80 protein-coding genes in *C. glandulosa* and 81 in other species, four rRNA genes and 31 tRNA genes (Figure 1 and Table 2). Eight protein-coding genes, four rRNA and seven tRNA were found in the IR regions. In the *C. glandulosa* species, the LSC region contained 61 protein-coding genes, whereas it included 62 in other species and 23 tRNA genes; the remaining 12 protein-coding genes and 1 tRNA are situated in the SSC region.

Some protein-coding genes and tRNA genes in the chloroplast genome of angiosperms contain an intron [49,50], as is found in the plastomes of the four species (*C. farinosa*, *C. glandulosa*, *M. crassifolia* and *M. oblongifolia*). In the total genes of the cp genomes (out of the total genes in all chloroplast genomes), 13 genes in *C. glandulosa* and *M. crassifolia* and 14 genes in *C. farinosa* and *M. oblongifolia* include an intron; some genes are protein-coding genes (nine in *C. farinosa* and *M. oblongifolia* and eight in *C. glandulosa* and *M. crassifolia*) while the remaining five are tRNA genes. Four genes (*rpl2*, *ndhB*, *trnI-GAU* and *trnA-UGC*) that have introns are situated in the inverted repeat region, *ndhA* is located in the SSC region and the remainder is found in the LSC region. All genes have only one intron and only two genes, namely *ycf3* and *clpP,* have two introns. The gene *trnK*-*UUU* has the longest intron of 2542–2571 bp; this is a result of the *matK* gene being located within the intron of the gene.

### 2.2. Codon Usage

**Figure 2:**
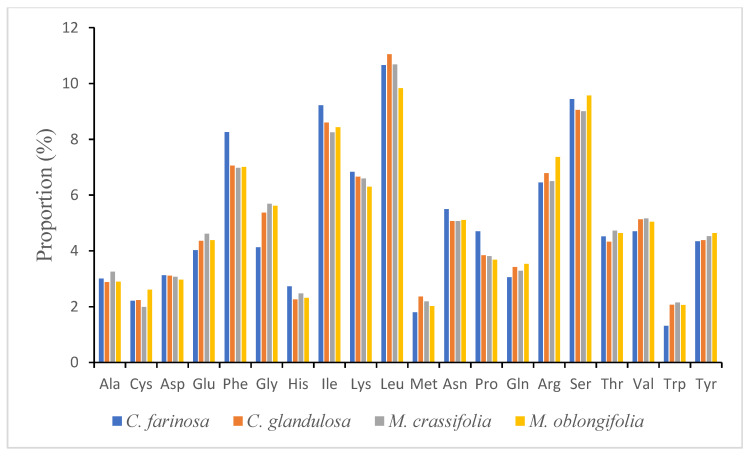
Amino acid frequencies in the four Capparaceae chloroplast genomes’ protein-coding sequences.

One of the factors shaping the evolution of the chloroplast genome is codon usage, which occurs as a result of bias in mutation [51]. The codon usage bias in the plastome was computed using the protein-coding genes and tRNA gene nucleotide sequences—104,575 bp, 106,488 bp, 105,750 bp and 99,100 bp for *C. farinosa, C. glandulosa, M. crassifolia* and *M. oblongifolia*, respectively. Supplementary Tables S1–S4 present the relative synonymous codon usage (RSCU) of each codon in the genome; these results suggested that all the genes are encoded by 33,686 codons in *C. farinosa,* 34,303 codons in *C. glandulosa*, 34,064 codons in *M. crassifolia* and 31,920 codons in *M. oblongifolia*. The most frequently occurring codons are for the amino acids leucine 3290 (9.76%), 3599 (10.49%), 3452 (10.13%) and 2951 (9.24%), respectively (Figure 2), which have been found in other flowering plant genomes [52], whereas methionine has the fewest codons in the genome at 620 (1.84%) in *C. farinosa* and 606 (1.89%) in *M. oblongifolia*, and for tryptophan it is 674 (1.96%) in *C. glandulosa* and 695 (2.04%) in *M. crassifolia*. A- and T- endings were discovered to be less frequent than their counterparts G- and C-. The codon usage bias is low in the cp genomes of Capparaceae species (Tables S1–S4). Additionally, the results show that the RSCU values of 27 codons were >1, all with A/T- endings, whereas the other 28 codons were <1, and all ended with G/C. The RSCU values of tryptophan and methionine amino acids are 1, so they are the only amino acids with no codon bias.

### 2.3. RNA Editing Sites

RNA editing features a set of processes that comprise of insertion, deletion or modifications of nucleotides that alter the DNA-encoded sequence [53], which represents a way to create transcript and protein diversity [54]. Some chloroplast RNA editing sites are preserved in plants [55]. The RNA editing sites in the *C. farinosa, C. glandulosa, M. crassifolia* and *M. oblongifolia* chloroplast genomes were predicted using the PREP suite program; the first codon position of the first nucleotide was used in all of the analyses. The results show that conversion of the amino acid serine into leucine was the majority of the conversions in the codon positions (Tables S5–S8). This conversion is found to occur more frequently [56]. In total, 48 editing sites in the genus *Maerua* and 50 in the genus *Cadaba* were revealed by the program. Twenty protein-coding genes in *C. farinosa* and 19 protein-coding genes in *C. glandulosa*, *M. crassifolia* and *M. oblongifolia* were distributed across the editing sites. As stated in previous studies [57,58,59], the *ndhB* genes have the largest number of editing sites (nine sites), followed by *ndhD* (nine sites in *C. farinosa* and *M. oblongifolia* and eight sites in *C. glandulosa* and *M. crassifolia*), while *accD, atpF*, *ccsA, clpP, PsaI, psbG, psbF, rpoA, rpl20, rps2 and rps16* have at least one site each. Certain RNA sites, amidst all the conversions in the RNA editing (modification) sites, changed the amino acid from proline to serine. RNA-predicting sites in the first codon of the first nucleotides are not present in the following genes: *atpA, atpB, atpI, ccsA* (only in *C. glandulosa*), *petB, petD, petG, petL, psaB, psbB, psbL, rpl2, rpl20* (except in *M. oblongifolia*), *rpl23, rps8 and ycf3,* among others. This result indicated that the preservation of RNA editing is fundamental [60,61].

### 2.4. Repeat Analysis

#### 2.4.1. Long Repeats

**Figure 3:**
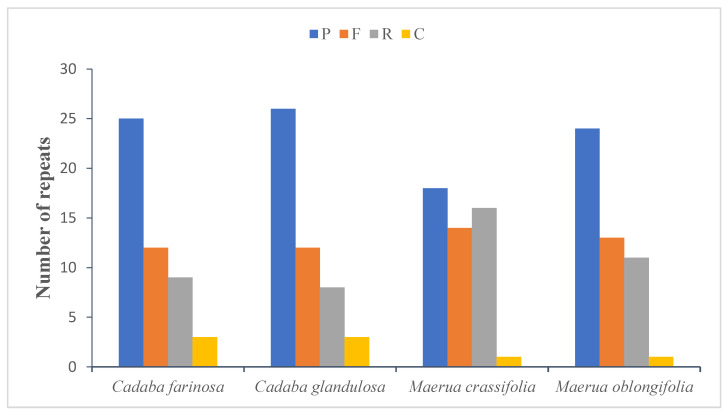
Number of different repeats in four chloroplast genomes of four species of Capparaceae. *p* = palindromic, F = forward, R = reverse and C= complement.

Repeat sequences in the chloroplast genomes of the four Capparaceae species were determined by the REPuter program with default settings; the obtained results clearly show that forward, reverse, palindrome and complemented repeats were detected in the cp genomes (Figure 3). The long repeat analysis in *C. farinosa*, *C. glandulosa, M. crassifolia* and *M. oblongifolia* showed 25–26–18–24 palindromic repeats, 12–12–14–13 forward repeats, 9–8–16–11 reverse repeats and 3–3–1–1 complement repeats, respectively (Figure 3 and Tables S9–S12). For the majority of the repeats, their sizes are: In *C. farinosa*—20–29 bp (69.38%), followed by 10–19 bp (22.44%), followed by 30–39 bp (4.08%), whereas 40–49 bp and 60–69 bp are the least common, at 2.04%. In *C. glandulosa*—20–29 bp (87.75%), followed by 30–39 bp (6.12%), whereas 10–19 bp, 40–49 bp and 60–69 bp are the least common, at 2.04%. In *M. crassifolia*—20–29 bp (48.97%), followed by 10–19 bp (38.77%), with 50–59 bp and 40–49 bp being the least common, at 6.12% and 4.08%, respectively, whereas 30–39 bp was at 2.04%. In *M. oblongifolia*—20–29 bp (65.30%), followed by 10–19 bp (26.53%), followed by 50–59 bp (4.08%), whereas 30–39 bp and 40–49 bp are the least common, at 2.04%. In total, there are 49 repeats in the chloroplast genomes of the four species. In the first location, the codon region harbored 42.85% of the repeats in *C. farinosa, M. crassifolia* and *M. oblongifolia* and 34.69% in *C. glandulosa;* tRNA contained 7 repeats (14.28%) in *C. farinosa*, 8 repeats (16.32%) in *C. glandulosa*, 9 repeats (18.36%) in *M. crassifolia* and 10 repeats (20.40%) in *M. oblongifolia;* the remainder of the repeats are located in the protein-coding genes—7 repeats (14.28%) in *C. farinosa* and *C. glandulosa,* 6 repeats (12.24%) in *M. crassifolia* and 12 repeats (24.48%) in *M. oblongifolia.* The length of repeated sequences in the four Capparaceae chloroplast genomes ranged from 10 to 59 bp, analogous to the lengths in other angiosperm plants [62,63,64].

#### 2.4.2. Simple Sequence Repeats (SSRs)

**Figure 4:**
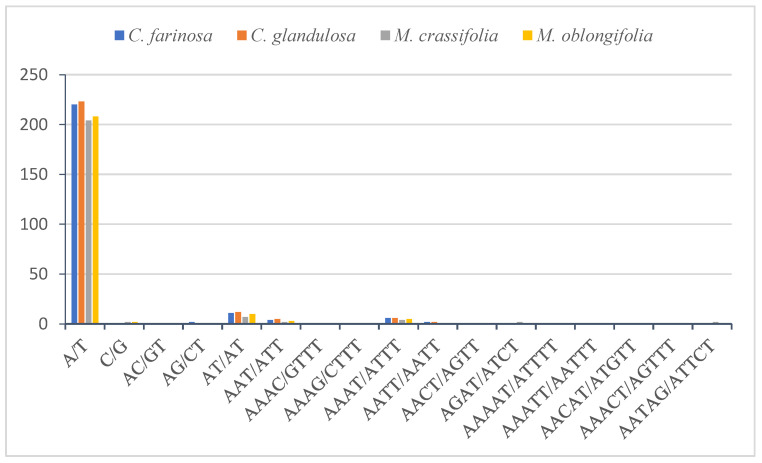
Frequency of different SSR motifs in different repeat types in *C. farinosa*, *C. glandulosa*, *M. crassifolia* and *M. oblongifolia* chloroplast genomes.

The SSRs or microsatellites are a group of short repeat sequences of nucleotide series (1–6 bp), which are used as a tool to facilitate the assessment of molecular diversity [65]. The genetic variation within and among species with the valuable molecular marker of the SSRs is extremely important for studying genetic heterogeneity and contributes to species recognition [66,67,68]. In this study, there are 249 microsatellites found in the plastid genome of *C. farinosa*, in *C. glandulosa* there are 251, in *M. crassifolia* there are 227 and in *M. oblongifolia* there are 233 (Table 3). The majority of SSRs in the cp genome in *C. farinosa*, *C. glandulosa, M. crassifolia* and *M. oblongifolia* are mononucleotide (88.75%, 89.24%, 90.74% and 90.12%, respectively), of which most are poly T and A (Figure 4). Polythymine (poly T) constituted 50.60%, 52.19%, 51.98% and 52.78%, respectively, whereas polyadenine (poly A) constituted 37.75%, 36.65%, 37.88% and 36.48%, respectively. Only a single polycytosine (poly C) (0.40% and 0.42%) was present in *C. farinosa* and *M. oblongifolia,* whereas two (0.88%) were present in *M. crassifolia*, and only a single polyguanine (poly G) (0.39% and 0.42%) was present in *C. glandulosa* and *M. oblongifolia*. Among the dinucleotides, AT/AT, AC/GT and AG/CT were found in all genomes. Reflecting series complementary, only one trinucleotide, AAT/ATT, six tetranucleotides, AAAC/GTTT, AAAG/CTTT, AAAT/ATTT, AATT/AATT, AACT/AGTT and AGAT/ATCT, and five pentanucleotides, AAAAT/ATTTT, AAATT/AATTT, AACAT/ATGTT, AAACT/AGTTT and AATAG/ATTCT, were discovered in the genome, while no hexanucleotide repeat was present (Figure 4). A high richness in mononucleotides poly A and T has been observed in most flowering plants’ cp genomes [62].

**Figure 5:**
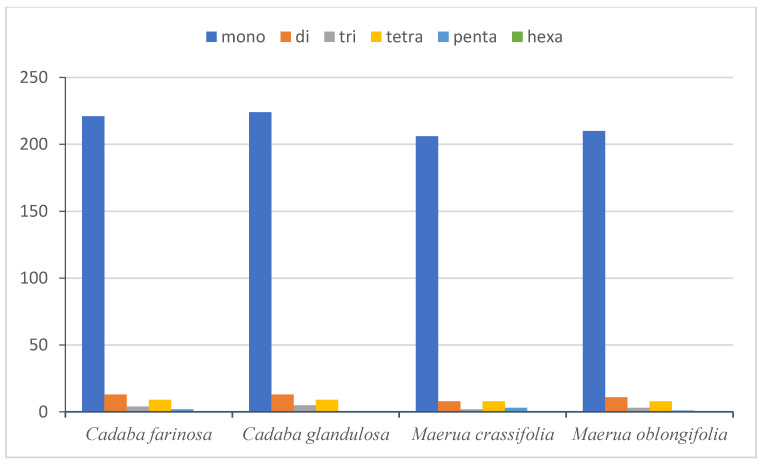
Number of different SSR types in the four chloroplast genomes of Capparaceae.

The comparison of simple sequence repeats between the chloroplast genomes of the four Capparaceae species (Figure 5) indicated that the more frequent occurrences are the mononucleotide repeats in all the genomes. The largest number of mononucleotides in *C. glandulosa* was 224, while it did not possess a pentanucleotide, like the remaining three species. Hexanucleotide was not present in any of the four species.

### 2.5. Comparative Analysis of the Capparaceae Species Cp Genome

**Figure 6:**
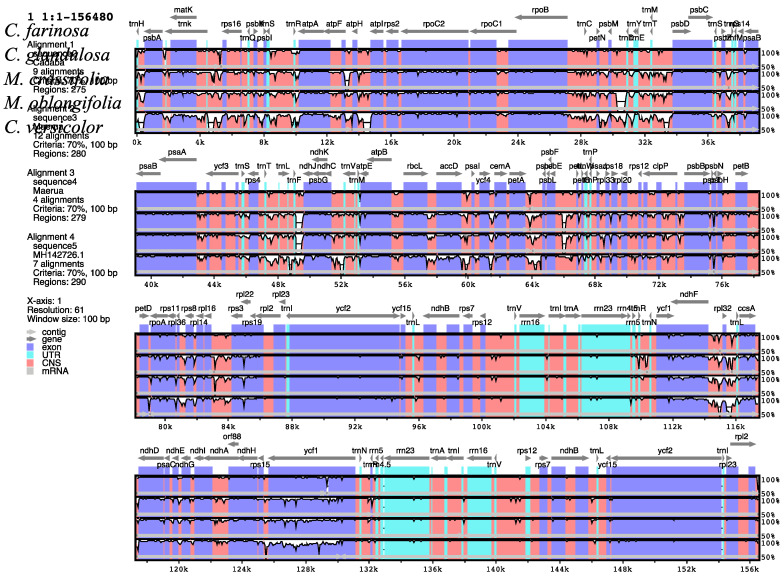
Alignment of chloroplast genomes of *C. farinosa*, *C. glandulosa*, *M. crassifolia, M. oblongifolia* and *C. versicolor* performed with *C. farinosa* as reference. Transcription direction is indicated by the gray arrows at the top, protein coding is represented by blue bars, non-coding sequence CNS is represented by pink bars and tRNAs and rRNAs are represented by light green. The cp genome is identified by the coordinates in the x-axis, while the y-axis represents the percentage identity within 50–100%.

To analyze the DNA sequence divergence in the chloroplast genomes of the five species of Capparaceae, a comparative analysis was done using the mVISTA program to align the sequences. Sequence alignment was conducted among four chloroplast genomes of Capparaceae and compared with the chloroplast genome of *Capparis versicolor* (MH142726), available in GenBank. To understand the structural characteristics in the cp genomes, the annotation of *C. farinosa* was used as a reference. The alignment outcome reveals highly conserved genomes with few variations. As in most chloroplast genomes of angiosperm plants, non-coding counterparts were conserved less than the gene-coding regions (Figure 6). Among the five cp genomes, the results showed that *trnH*(*GUG*)-*psbA*, *rps16*-*trnQ*, *psbI-trnS, trnS-trnR, petN-psbM, psbM-trnD, trnE-trnT, trnS-trnG, trnT-trnL, trnF-ndhJ, rbcL-accD, psbE-petL, rbs16-rbs3* and *ndhF-rpl32* were the most divergent non-coding regions. However, it was detected that some variations occurred in the following genes: *atpF, rpoC2, rps19* and *ycf1*.

**Figure 7:**
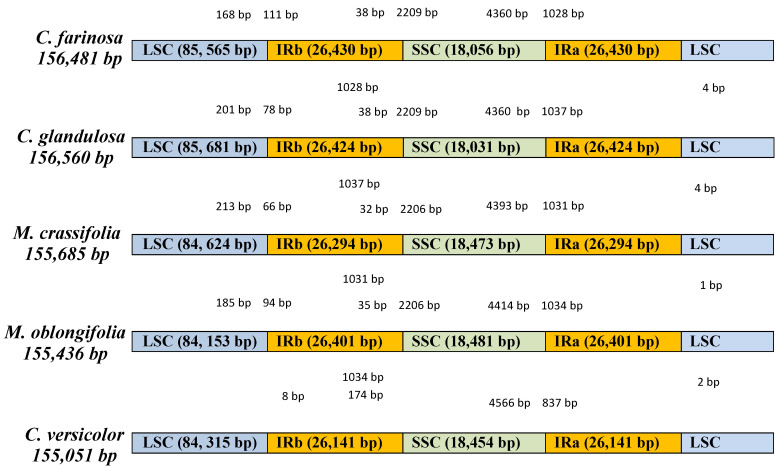
Comparison of the IR, SSC and LSC junction positions among five chloroplast genomes of Capparaceae.

Although angiosperms retain the structure and size of the chloroplast genome [68], some evolutionary events occur in the genome, such as expansion and contraction, that alter the size of the genome and the boundaries of the LSC, SSC, IRa and IRb regions [69,70]. We compared between IR-LCS and IR-SSC the boundaries of the five cp genomes of Capparaceae (*Cadaba farinosa*, *Cadaba glandulosa*, *Maerua crassifolia*, *Maerua oblongifolia* and *Capparis versicolor*) and the result presented a similarity among the compared plastomes of *Cadaba* and *Maerua* species, with a slight variation among *C. versicolor* (Figure 7). The chloroplast genome of *C. versicolor* (155,051 bp) was the smallest, whereas the genome of *C. glandulosa* (156,560 bp) was the largest. The smallest IR region is in *C. versicolor* (26,141 bp). The lengths of LSC regions varied among the five Capparaceae species (85,565 bp, 85,681 bp, 84,624 bp, 84,153 bp, 84,315 bp, respectively). The location of the *rpsl9* gene is between the junction of the LSC and IRb regions in five species and is in the LSC region in *C. versicolor*. The *ycf1* gene is located in IRb regions, except in *C. versicolor*, and it crosses the SSC/IRa region and extends by different lengths into the SSC region within the genome (*C. farinosa* and *C. glandulosa* 4360 bp; *M. crassifolia* 4393 bp; *M. oblongifolia* 4414 bp and *C. versicolor* 4566 bp). The *ndhF* gene is found in the IRb/SSC and is 38 bp in *C. farinosa* and *C. glandulosa*, 32 bp in *M. crassifolia* and 35 bp in *M. oblongifolia* in the IRb region, and it extends into the SSC region by 2209 bp in *C. farinosa* and *C. glandulosa* and 2206 bp in *M. crassifolia* and *M. oblongifolia*, and is 174 bp away from the border in the *C. versicolor* genome.

### 2.6. Divergence of Protein-Coding Gene Sequence

**Figure 8:**
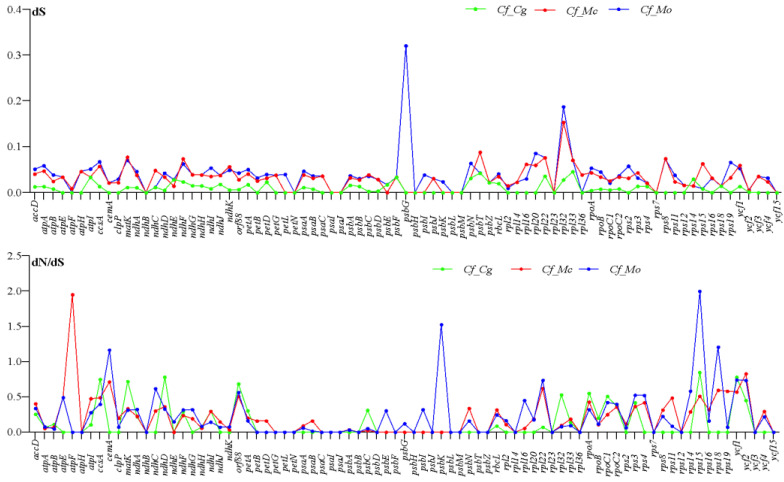
The synonymous (dS) and dN/dS ratio values of 81 protein-coding genes from four Capparaceae cp genomes.

The cp genomes of four Capparaceae species include 80 protein-coding genes in *C. glandulosa* and 81 genes in other species. To detect the genes under selective pressure, the rates of synonymous (dS) and non-synonymous (dN) substitution and dN/dS ratio were calculated. The results showed that in all of the paired genes of *C. farinosa* vs. *C. glandulosa,* the dN/dS ratio is less than 1, and most of the paired genes are less than 1 except *atpF* in *C. farinosa* vs. *M. crassifolia* and *cemA, psbK* and *rps18* in *C. farinosa* vs. *M. oblongifolia,* having values of 1.16, 1.52 and 1.2, respectively (Figure 8). The result of the dN/dS ratio obtained in this study is consistent with other related studies [52,53]. In all the genes, the synonymous (dS) values range from 0 to 0.32 (Figure 8).

### 2.7. Phylogenetic Analysis

**Figure 9:**
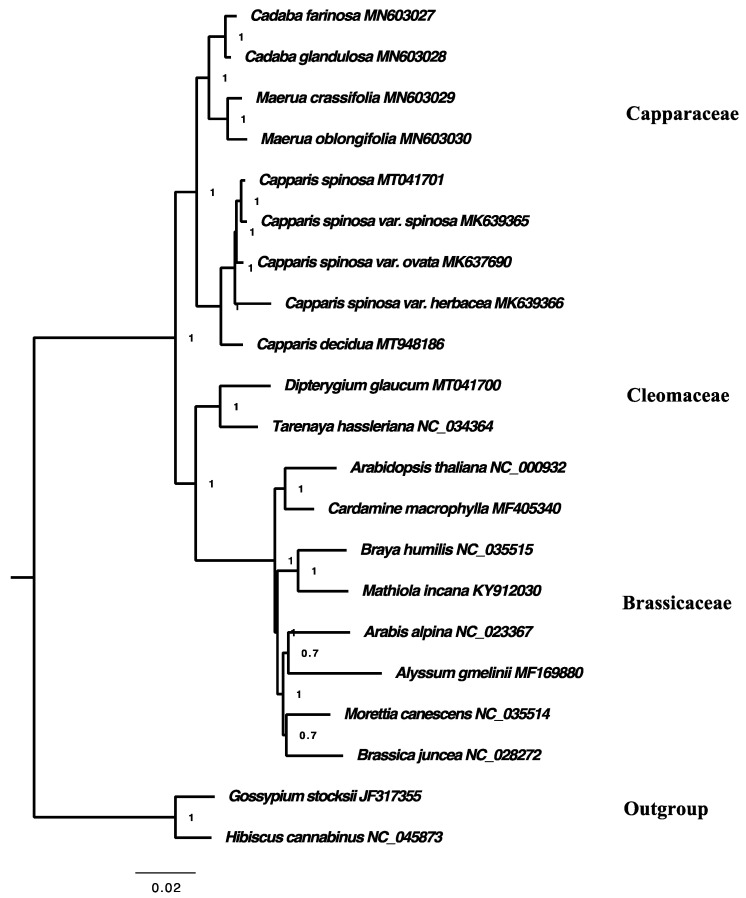
Phylogenetic tree reconstruction based on the complete chloroplast genome of 21 taxa inferred from Bayesian inference (BI) methods, showing relationships within Brassicales. Numbers in the clade represent posterior probability (PP) values.

Phylogenetic relationships based on Bayesian analysis and maximum parsimony were congruent and placed all samples into three main clades, with strong support in all the nodes with PP 1.00 (Figure 9). The first clade contains species of the Capparaceae family and is divided into two subclades; the first subclade includes species of genera *Cadaba* and *Maerua*, while the second clade includes species of genus *Capparis*. The second clade comprises Cleomaceae species, while the third clade includes species from the Brassicaceae family. The phylogenetic tree showed that the Capparaceae family is the earliest diverging lineage among the three families and is sister to Cleomaceae and Brassicaceae. It is clear in this phylogenetic result that Cleomaceae was separated from Capparaceae and became a sister to the Brassicaceae family, as reported by [19,20]; this is consistent with some previous classifications of the order Brassicales.

## 3. Materials and Methods

### 3.1. Plant Material and DNA Extraction

Fresh young leaves were collected in 2018 during field investigations in Saudi Arabia: *C. farinosa* in Jeddah (21°26′45.2″ N 39°25′22.9″ E) on 21 April, *C. glandulosa* in Jeddah (21°26′45.3″ N 39°25′22.9″ E) on 21 April, *M. crassifolia* in Makkah (21°13′17.4″ N 39°49′36.1″ E) on 28 April and *M. oblongifolia* in Jeddah (21°28′31.7″ N 39°50′36.8″ E) on 21 April. No permission was required to collect the plant samples. Species were identified and verified by Dr. Dhafer Alzahrani, Department of Biological Sciences, Faculty of Sciences, King Abdulaziz University, Jeddah, Saudi Arabia. A voucher specimen was prepared and deposited in the herbarium of King Abdulaziz University, Jeddah with the accession numbers: *C. farinosa* (KAU27480), *C. glandulosa* (KAU27481) *M. crassifolia* (KAU27482), *M. oblongifolia* (KAU27483). Total genomic DNA was extracted from the samples using the Qiagen genomic DNA extraction kit, according to the manufacturer’s protocols. 

### 3.2. Library Construction, Sequencing and Assembly

Input material for the DNA sample preparations was derived (or taken) from a total amount of 1.0 μg DNA. The NEBNext DNA Library Prep Kit was used to generate sequence libraries according to the manufacturer’s recommendation; indices were also added to each sample. Genomic DNA was randomly fragmented by shearing to a size of 350 bases in length. The ends of randomly fragmented DNA were repaired and A-tailed, adapters were ligated with NEBNext for Illumina sequencing, then the PCR improved by P5 and indexed P7 oligo sequences. The AMPure XP system was used to purify the PCR products; subsequent findings were analyzed by the Agilent 2100 Bioanalyzer for size distribution and later quantified using real-time PCR. After pooling, the qualified libraries were fed into an Illumina HiSeq 2500 system (350 bp paired ends reads); this was based on its effective concentration and expected data volume. The raw reads (19,844,190 bp, 19,053,503 bp, 19,440,639 bp and 19,929,468 bp for *C. farinosa, C. glandulosa, M. crassifolia* and *M. oblongifolia*, respectively) were cleaned reads (5 Gb) to remove low-quality sequences and adaptors; they were then filtered for PCR duplicates using PRINSEQlite v0.20.4 [71]. The clean raw reads were subjected to de novo assembly from the whole genome sequences using NOVOPlasty 2.7.2 [72] with kmer (K-mer = 31–33). The *trnH-psbA* of *Cadaba farinosa* (KR735837.1) was used as a seed and the complete plastome of *Arabidopsis thaliana* (KX551970.1) was used as a reference for the assembly of the *Cadaba farinosa* cp genome. The assembled cp genome of *Cadaba farinosa* was used as seed and reference for the assembly of the *Cadaba glandulosa* plastome. For *Maerua crassifolia, the rpoC1* gene of *M. crassifolia* (JQ845894.1) was used as seed and the complete cp genome of *C. farinosa* was used as reference. The assembled cp genome of *M. crassifolia* was used as seed and reference for the assembly of *M. oblongifolia*. Finally, each species generated one contig that contained the complete chloroplast genome sequence.

### 3.3. Gene Annotation

Genes were annotated using the Dual Organellar GenoMe Annotator (DOGMA, University of Texas at Austin, Austin, TX, USA) [73]. The positions of start and stop codons were adjusted manually. tRNA genes were identified by the trnAscan-SE server (http://lowelab.ucsc.edu/tRNAscan-SE/ (accessed on 20 June 2019) [74]. Organellar Genome DRAW (OGDRAW) [75] was used to draw the genome maps.

### 3.4. Sequence Analysis

MEGA 6.0 was used to compute the codon usage, base composition, and the relative synonymous codon usage values (RSCUs). The RNA editing sites in cp protein-coding genes of the Capparaceae species were predicted using PREP suite [76] with a 0.8 cutoff value. Simple sequence repeats (SSRs) were identified in the chloroplast genomes of the four species (*C. farinosa, C. glandulosa, M. crassifolia* and *M. oblongifolia*) using the online software MIcroSAtellite (MISA) [77] with the following parameters set: eight, five, four and three repeat units for mononucleotides, dinucleotides, trinucleotides and tetra-, penta-, hexanucleotide SSR motifs, respectively. To identify the size and location of long repeats (palindromic, forward, reverse and complement) in the four species of Capparaceae being studied, the online program REPuter (https://bibiserv.cebitec.uni-bielefeld.de/reputer (accessed on 22 June 2019) [76], with standard settings, was used.

### 3.5. Genome Comparison

The chloroplast genomes of *C. farinosa, C. glandulosa, M. crassifolia* and *M. oblongifolia* were compared using the program mVISTA [78], and the annotation of *C. farinosa* was used as a reference in the Shuffle-LAGAN mode [79]. The four species of Capparaceae were compared against the border region between inverted repeat (IR), large single copy (LSC) and small single copy (SSC).

### 3.6. Characterization of Substitution Rate

To detect the genes that are under selection pressure, the substitution rate of the synonymous (dS) and non-synonymous (dN) substitution and the dN/dS ratio were analyzed using DNAsp v5.10.01 [80], the cp genome of *C. farinosa* was compared with the cp genome of *C. glandulosa*, *M. crassifolia* and *M. oblongifolia*. Separate protein-coding genes were aligned individually using Geneious version 8.1.3 software, while the protein sequence was translated from aligned sequences.

### 3.7. Phylogenetic Analysis

The analysis was conducted based on the complete chloroplast genome sequences of nine Capparaceae taxa, six species and three varieties, *C. farinosa,* MN603027, *C. glandulosa*, MN603028, *M. crassifolia,* MN603029, *M. oblongifolia,* MN603030, *Capparis spinosa*, MT041701, *Capparis spinosa* var. *spinosa*, MK639365, *Capparis spinosa* var. *herbacea*, MK639366, *Capparis spinosa* var. *ovata*, MK637690 and *Capparis decidua* MT948186, two Cloemaceae species, eight species of Brassicaceae and two species of Malvaceae, as an outgroup. All sequences were aligned using MAFFT software [81] with default settings. The phylogenetic trees were reconstructed based on maximum parsimony analysis using PAUP software (version 4.0b10) [82], utilizing tree bisection and reconnection branch swapping, with MulTrees on, saving a maximum of 1000 trees per replicate. Missing characters were treated as gaps. The bootstrap analysis confidence was based on 1000 replicates. MrBayes version 3.2.6 [83] was used to conduct Bayesian analysis and jModelTest version 3.7 [84] was used to select the appropriate model.

## 4. Conclusions

This current study used the Illumina HiSeq 2500 platform to obtain the first complete chloroplast sequences of four medicinal Capparaceae species: *C. farinosa*, *C. glandulosa*, *M. crassifolia* and *M. oblongifolia*. The four species are divided into two groups: *C. farinosa* and *C. glandulosa* belong to the tribe Cadabeae; *M. crassifolia* and *M. oblongifolia* belong to the tribe Maerueae. This study can be used to accurately identify species during different medicinal uses based on their plastid genome. 

## Figures and Tables

**Table 1:** Base content in the *C. farinosa*, *C. glandulosa*, *M. crassifolia* and *M. oblongifolia* chloroplast genomes.

Species	*C. farinosa*	*C. glandulosa*	*M. crassifolia*	*M. oblongifolia*
Genome size (bp)	156,481	156,560	155,685	155,436
IR (bp)	26,430	26,424	26,294	26,401
LSC (bp)	85,565	85,681	84,624	84,153
SSC (bp)	18,056	18,031	18,473	18,481
Total number of genes	138	137	138	138
rRNA	4	4	4	4
tRNA	31	31	31	31
Protein-coding genes	81	80	81	81
A%	31	31	31	31
C%	18	18	18	18
T%	32	32	32	32
G%	17	17	17	17

**Table 2:** Gene contents in the chloroplast genomes of *Cadaba* and *Maerua* species.

Category	Gene Groups	Gene Names
RNA genes	Ribosomal RNA genes (rRNA)	*rrn5, rrn4.5, rrn16, rrn23*
	Transfer RNA genes (tRNA)	*trnH-GUG, trnK-UUU*^+^*, trnQ-UUG, trnS-GCU, trnS-CGA*^+^*, trnR-UCU, trnC-GCA, trnD-GUC, trnY-GUA, trnE-UUC, trnT-GGU, trnS-UGA, trnG-GCC, trnfM-CAU, trnS-GGA, trnT-UGU, trnL-UAA*^+^*, trnF-GAA, trnV-UAC*^+^*, trnM-CAU, trnW-CCA, trnP-UGG, trnP-GGG, trnI-CAU*^a^*, trnL-CAA*^a^*, trnV-GAC*^a^*, trnI-GAU*^+,a^*, trnA-UGC*^+,a^*, trnR-ACG*^a^, *trnN-GUU*^a^, *trnL-UAG*
Ribosomal proteins	Small ribosomal subunit	*rps2, rps3, rps4, rps7* ^a^ *, rps8, rps11, rps12* ^a^ *, rps14, rps15, rps16* ^+^ *, rps18, rps19*
Transcription	Large ribosomal subunit	*rpl2* ^+,a^ *, rpl14, rpl16, rpl20, rpl22, rpl23* ^a^ *, rpl32, rpl33, rpl36*
	DNA dependent RNA polymerase	*rpoA, rpoB, rpoC1* ^+^ *, rpoC2*
Protein-coding genes	Photosystem I	*psaA, psaB, psaC, psaI, psaJ, ycf3* ^++^
	Photosystem II	*psbA, psbB, psbC, psbD, psbE, psbF, psbG, psbH, psbI, psbJ, psbK, psbL, psbM, psbN, psbT, psbZ, ndhK* *
	Subunit of cytochrome	*petA, petB, petD, petG, petL, petN*
	Subunit of synthase	*atpA, atpB, atpE, atpF* ^+^ *, atpH, atpI*
	Large subunit of Rubisco	*rbcL*
	NADH dehydrogenase	*ndhA* ^+^ *, ndhB* ^+,a^ *, ndhC, ndhD, ndhE, ndhF, ndhG, ndhH, ndhI, ndhJ*
Other genes	ATP dependent protease subunit P	*clpP* ^++^
	Chloroplast envelope membrane protein	*cemA*
	Maturase	*matK*
	Subunit of acetyl-CoA carboxylase	*accD*
	C-type cytochrome synthesis	*ccsA*
	Translation initiation factor	*infA*
	Hypothetical proteins	*ycf2* ^a^ *, ycf4, ycf15* ^a^
	Component of the TIC complex	*ycf1* ^a^

+ Gene with one intron, ++ gene with two introns and a gene with multiple copies. ^a^ gene with multiple copies. * *ndhK* in group photosystem II in *C. farinosa* and group NADH dehydrogenase in *C. glandulosa, M. crassifolia* and *M. oblongifolia.*

**Table 3:** Simple sequence repeats in the *C. farinosa*, *C. glandulosa*, *M. crassifolia* and *M. oblongifolia* chloroplast genomes.

SSR Type	Repeat Unit	Species			
		*C. farinosa*	*C. glandulosa*	*M. crassifolia*	*M. oblongifolia*
Mono	A/T	220	223	204	208
	C/G	1	1	2	2
Di	AC/GT	0	1	0	0
	AG/CT	2	0	1	1
	AT/AT	11	12	7	10
Tri	AAT/ATT	4	5	2	3
Tetra	AAAC/GTTT	0	0	1	0
	AAAG/CTTT	0	0	0	1
	AAAT/ATTT	6	6	4	5
	AATT/AATT	2	2	1	0
	AACT/AGTT	0	0	0	1
	AGAT/ATCT	1	1	2	1
Penta	AAAAT/ATTTT	1	0	0	0
	AAATT/AATTT	0	0	1	0
	AACAT/ATGTT	0	0	0	1
	AAACT/AGTTT	1	0	0	0
	AATAG/ATTCT	0	0	2	0

## Data Availability

The complete chloroplast genome sequence of four Capparaceae chloroplast genome sequences were deposited in GenBank at https://www.ncbi.nlm.nih.gov, (accession numbers: *C. farinosa*, MN603027; *C. glandulosa*, MN603028; *M. crassifolia*, MN603029 and *M. oblongifolia*, MN603030).

## Supplementary Materials

The following are available online at https://www.mdpi.com/article/10.3390/plants10061229/s1, Table S1: Codon–anticodon recognition patterns and codon usage of the *Cadaba farinosa* chloroplast genome; Table S2: Codon–anticodon recognition patterns and codon usage of the *Cadaba glandulosa* chloroplast genome; Table S3: Codon–anticodon recognition patterns and codon usage of the *Maerua crassifolia* chloroplast genome; Table S4: Codon–anticodon recognition patterns and codon usage of the *Maerua oblongifolia* chloroplast genome; Table S5: Predicted RNA editing site in the *Cadaba farinosa* chloroplast genome; Table S6: Predicted RNA editing site in the *Cadaba glandulosa* chloroplast genome; Table S7: Predicted RNA editing site in the *Maerua crassifolia* chloroplast genome; Table S8: Predicted RNA editing site in the *Maerua oblongifolia* chloroplast genome; Table S9: Repeat sequences present in the *Cadaba farinosa* chloroplast genome; Table S10: Repeat sequences present in the *Cadaba glandulosa* chloroplast genome; Table S11: Repeat sequences present in the *Maerua crassifolia* chloroplast genome; Table S12: Repeat sequences present in the *Maerua oblongifolia* chloroplast genome.
