# Supplementary material for: Comparative Analysis of Chloroplast Genomes of Four Medicinal Capparaceae Species: Genome Structures, Phylogenetic Relationships and Adaptive Evolution
Source: Plants (Basel). 2021 Jun 17;10(6):1229. doi: 10.3390/plants10061229 (PMC8234754; doi:10.3390/plants10061229)
Supplement: Supplementary file 1 [file plants-10-01229-s001.zip › plants-1151648-supplementary.pdf]

Supplementary

Title: **Comparative Analysis of Chloroplast Genomes for four Medicinal Capparaceae Species: Molecular Structure, Phylogenetic Relationships and Adaptive Evolution**

Dhafer A. Alzahrani [1,*], Enas J. Albokhari [1,2], Samaila S. Yaradua [1] and Abidina Abba [1]

1 Department of Biological Sciences, Faculty of Science, King Abdulaziz University, P.O. Box 80203, Jeddah 21589, Saudi Arabia

2 Department of Biological Sciences, Faculty of Applied Sciences, Umm Al-Qura University, Makkah, Saudi Arabia

* Correspondence: **Dhafer A. Alzahrani** dalzahrani@kau.edu.sa**, Tel: 00966555546409; Enas J. Albokhari** enasbokhary@hotmail.com

Table S1. Codon – anticodon recognition patterns and codon usage of the *Cadaba farinosa* chloroplast genome

Codon	Amino Acid	RSCU	tRNA	Codon	Amino Acid	RSCU	tRNA
UUU	Phe	1.24	*trnF-GAA*	UAU	Tyr	1.31	*trnY-GUA*
UUC	Phe	0.76		UAC	Tyr	0.69	
UUA	Leu	1.43	*trnL-UAA*	UAA	Stop	1.1	
UUG	Leu	1.36	*trnL-CAA*	UAG	Stop	0.84	
CUU	Leu	1.22	*trnL-UAG*	CAU	His	1.38	*trnH-GUG*
CUC	Leu	0.67		CAC	His	0.62	
CUA	Leu	0.79		CAA	Gln	1.36	*trnQ-UUG*
CUG	Leu	0.53		CAG	Gln	0.64	
AUU	Ile	1.34	*trnI-GAU*	AAU	Asn	1.33	*trnN-GUU*
AUC	Ile	0.82		AAC	Asn	0.67	
AUA	Ile	0.85	*trnI-CAU*	AAA	Lys	1.35	*trnK-UUU*
AUG	Met	1	*trnM-CAU*	AAG	Lys	0.65	
GUU	Val	1.38	*trnV-GAC*	GAU	Asp	1.41	*trnD-GUC*
GUC	Val	0.76		GAC	Asp	0.59	
GUA	Val	1.22		GAA	Glu	1.35	*trnE-UUC*
GUG	Val	0.64	*trnV-UAC*	GAG	Glu	0.65	
UCU	Ser	1.42	*trnS-GGA*	UGU	Cys	1.03	*trnC-GCA*
UCC	Ser	0.93		UGC	Cys	0.97	
UCA	Ser	1.38		UGA	Stop	1.06	
UCG	Ser	0.73	*trnS-UGA*	UGG	Trp	1	*trnW-CCA*
CCU	Pro	1.24	*trnP-UGG*	CGU	Arg	0.65	*trnR-ACG*
CCC	Pro	0.8		CGC	Arg	0.32	*trnR-UCU*
CCA	Pro	1.23		CGA	Arg	1.01	
CCG	Pro	0.73		CGG	Arg	0.64	
ACU	Thr	1.04		AGA	Arg	2.05	
ACC	Thr	0.95		AGG	Arg	1.33	
ACA	Thr	1.25	*trnT-GGU*	AGU	Ser	0.84	*trnS-GCU*
ACG	Thr	0.75	*trnT-UGU*	AGC	Ser	0.7	
GCU	Ala	1.34	*trnA-UGC*	GGU	Gly	1	
GCC	Ala	0.81		GGC	Gly	0.57	
GCA	Ala	1.14		GGA	Gly	1.44	
GCG	Ala	0.72		GGG	Gly	0.99	*trnG-UCC*

Table S2. Codon – anticodon recognition patterns and codon usage of the *Cadaba glandulosa* chloroplast genome

Codon	Amino Acid	RSCU	tRNA	Codon	Amino Acid	RSCU	tRNA
UUU	Phe	1.23	*trnF-GAA*	UAU	Tyr	1.36	*trnY-GUA*
UUC	Phe	0.77		UAC	Tyr	0.64	
UUA	Leu	1.27	*trnL-UAA*	UAA	Stop	1.07	
UUG	Leu	1.28	*trnL-CAA*	UAG	Stop	0.96	
CUU	Leu	1.19	*trnL-UAG*	CAU	His	1.27	*trnH-GUG*
CUC	Leu	0.66		CAC	His	0.73	
CUA	Leu	0.98		CAA	Gln	1.3	*trnQ-UUG*
CUG	Leu	0.63		CAG	Gln	0.7	
AUU	Ile	1.27	*trnI-GAU*	AAU	Asn	1.35	*trnN-GUU*
AUC	Ile	0.77		AAC	Asn	0.65	
AUA	Ile	0.97	*trnI-CAU*	AAA	Lys	1.34	*trnK-UUU*
AUG	Met	1	*trnM-CAU*	AAG	Lys	0.66	
GUU	Val	1.37	*trnV-GAC*	GAU	Asp	1.44	*trnD-GUC*
GUC	Val	0.71		GAC	Asp	0.56	
GUA	Val	1.17		GAA	Glu	1.36	*trnE-UUC*
GUG	Val	0.75	*trnV-UAC*	GAG	Glu	0.64	
UCU	Ser	1.35	*trnS-GGA*	UGU	Cys	1.19	*trnC-GCA*
UCC	Ser	0.93		UGC	Cys	0.81	
UCA	Ser	1.44		UGA	Stop	0.97	
UCG	Ser	0.79	*trnS-UGA*	UGG	Trp	1	*trnW-CCA*
CCU	Pro	1.22	*trnP-UGG*	CGU	Arg	0.65	*trnR-ACG*
CCC	Pro	0.77		CGC	Arg	0.39	*trnR-UCU*
CCA	Pro	1.18		CGA	Arg	0.97	
CCG	Pro	0.83		CGG	Arg	0.71	
ACU	Thr	1.2		AGA	Arg	2.06	
ACC	Thr	0.75		AGG	Arg	1.22	
ACA	Thr	1.35	*trnT-GGU*	AGU	Ser	0.87	*trnS-GCU*
ACG	Thr	0.71	*trnT-UGU*	AGC	Ser	0.63	
GCU	Ala	1.34	*trnA-UGC*	GGU	Gly	1.04	
GCC	Ala	0.73		GGC	Gly	0.51	
GCA	Ala	1.23		GGA	Gly	1.42	
GCG	Ala	0.7		GGG	Gly	1.02	*trnG-UCC*

Table S3. Codon – anticodon recognition patterns and codon usage of the *Maerua crassifolia* chloroplast genome

Codon	Amino Acid	RSCU	tRNA	Codon	Amino Acid	RSCU	tRNA
UUU	Phe	1.22	*trnF-GAA*	UAU	Tyr	1.38	*trnY-GUA*
UUC	Phe	0.78		UAC	Tyr	0.62	
UUA	Leu	1.41	*trnL-UAA*	UAA	Stop	1.07	
UUG	Leu	1.26	*trnL-CAA*	UAG	Stop	0.87	
CUU	Leu	1.15	*trnL-UAG*	CAU	His	1.38	*trnH-GUG*
CUC	Leu	0.68		CAC	His	0.62	
CUA	Leu	0.92		CAA	Gln	1.39	*trnQ-UUG*
CUG	Leu	0.57		CAG	Gln	0.61	
AUU	Ile	1.32	*trnI-GAU*	AAU	Asn	1.4	*trnN-GUU*
AUC	Ile	0.78		AAC	Asn	0.6	
AUA	Ile	0.9	*trnI-CAU*	AAA	Lys	1.35	*trnK-UUU*
AUG	Met	1	*trnM-CAU*	AAG	Lys	0.65	
GUU	Val	1.39	*trnV-GAC*	GAU	Asp	1.46	*trnD-GUC*
GUC	Val	0.71		GAC	Asp	0.54	
GUA	Val	1.24		GAA	Glu	1.37	*trnE-UUC*
GUG	Val	0.66	*trnV-UAC*	GAG	Glu	0.63	
UCU	Ser	1.4	*trnS-GGA*	UGU	Cys	1.17	*trnC-GCA*
UCC	Ser	0.91		UGC	Cys	0.83	
UCA	Ser	1.39		UGA	Stop	1.06	
UCG	Ser	0.78	*trnS-UGA*	UGG	Trp	1	*trnW-CCA*
CCU	Pro	1.3	*trnP-UGG*	CGU	Arg	0.74	*trnR-ACG*
CCC	Pro	0.73		CGC	Arg	0.32	*trnR-UCU*
CCA	Pro	1.17		CGA	Arg	1	
CCG	Pro	0.8		CGG	Arg	0.6	
ACU	Thr	1.19		AGA	Arg	2.16	
ACC	Thr	0.88		AGG	Arg	1.18	
ACA	Thr	1.28	*trnT-GGU*	AGU	Ser	0.93	*trnS-GCU*
ACG	Thr	0.65	*trnT-UGU*	AGC	Ser	0.59	
GCU	Ala	1.48	*trnA-UGC*	GGU	Gly	1.14	
GCC	Ala	0.66		GGC	Gly	0.49	
GCA	Ala	1.25		GGA	Gly	1.39	
GCG	Ala	0.61		GGG	Gly	0.98	*trnG-UCC*

Table S4. Codon – anticodon recognition patterns and codon usage of the *Maerua oblongifolia* chloroplast genome

Codon	Amino Acid	RSCU	tRNA	Codon	Amino Acid	RSCU	tRNA
UUU	Phe	1.25	*trnF-GAA*	UAU	Tyr	1.33	*trnY-GUA*
UUC	Phe	0.75		UAC	Tyr	0.67	
UUA	Leu	1.32	*trnL-UAA*	UAA	Stop	1.04	
UUG	Leu	1.26	*trnL-CAA*	UAG	Stop	0.89	
CUU	Leu	1.24	*trnL-UAG*	CAU	His	1.36	*trnH-GUG*
CUC	Leu	0.75		CAC	His	0.64	
CUA	Leu	0.84		CAA	Gln	1.38	*trnQ-UUG*
CUG	Leu	0.6		CAG	Gln	0.62	
AUU	Ile	1.38	*trnI-GAU*	AAU	Asn	1.37	*trnN-GUU*
AUC	Ile	0.84		AAC	Asn	0.63	
AUA	Ile	0.78	*trnI-CAU*	AAA	Lys	1.35	*trnK-UUU*
AUG	Met	1	*trnM-CAU*	AAG	Lys	0.65	
GUU	Val	1.41	*trnV-GAC*	GAU	Asp	1.44	*trnD-GUC*
GUC	Val	0.73		GAC	Asp	0.56	
GUA	Val	1.13		GAA	Glu	1.35	*trnE-UUC*
GUG	Val	0.73	*trnV-UAC*	GAG	Glu	0.65	
UCU	Ser	1.48	*trnS-GGA*	UGU	Cys	1.14	*trnC-GCA*
UCC	Ser	0.94		UGC	Cys	0.86	
UCA	Ser	1.36		UGA	Stop	1.06	
UCG	Ser	0.75	*trnS-UGA*	UGG	Trp	1	*trnW-CCA*
CCU	Pro	1.23	*trnP-UGG*	CGU	Arg	0.64	*trnR-ACG*
CCC	Pro	0.85		CGC	Arg	0.35	*trnR-UCU*
CCA	Pro	1.18		CGA	Arg	0.98	
CCG	Pro	0.74		CGG	Arg	0.74	
ACU	Thr	1.12		AGA	Arg	2.05	
ACC	Thr	0.91		AGG	Arg	1.24	
ACA	Thr	1.27	*trnT-GGU*	AGU	Ser	0.81	*trnS-GCU*
ACG	Thr	0.7	*trnT-UGU*	AGC	Ser	0.65	
GCU	Ala	1.41	*trnA-UGC*	GGU	Gly	0.97	
GCC	Ala	0.74		GGC	Gly	0.56	
GCA	Ala	1.11		GGA	Gly	1.46	
GCG	Ala	0.74		GGG	Gly	1.01	*trnG-UCC*

Table S5. Predicted RNA editing site in the *Cadaba farinosa* chloroplast genome

Gene	Nucleotide Position	Amino Acid Position	Codon	Amino Acid	Score
accD	800	267	TCG=>TTG	S=>L	0.8
atpF	92	31	CCA=>CTA	P=>L	0.86
ccsA	880	294	CCT=>TCT	P=>S	0.86
clpP	562	188	CAT=>TAT	H=>Y	1
matK	706	236	CAT=>TAT	H=>Y	1
matK	1250	417	TCA=>TTA	S=>L	0.86
matK	1309	437	CAC=>TAC	H=>Y	1
ndhA	8	3	ACT=>ATT	T=>I	1
ndhA	125	42	ACA=>ATA	T=>I	0.8
ndhA	341	114	TCA=>TTA	S=>L	1
ndhB	155	52	TCA=>TTA	S=>L	1
ndhB	473	158	CCA=>CTA	P=>L	1
ndhB	592	198	CAT=>TAT	H=>Y	1
ndhB	617	206	TCA=>TTA	S=>L	0.8
ndhB	752	251	TCT=>TTT	S=>F	1
ndhB	836	279	TCA=>TTA	S=>L	1
ndhB	842	281	TCA=>TTA	S=>L	1
ndhB	1261	421	CAT=>TAT	H=>Y	1
ndhB	1487	496	CCA=>CTA	P=>L	1
ndhD	20	7	ACG=>ATG	T=>M	1
ndhD	65	22	TCT=>TTT	S=>F	0.8
ndhD	401	134	TCA=>TTA	S=>L	1
ndhD	692	231	TCA=>TTA	S=>L	1
ndhD	896	299	TCA=>TTA	S=>L	1
ndhD	905	302	CCT=>CTT	P=>L	1
ndhD	1316	439	TCA=>TTA	S=>L	0.8
ndhD	1328	443	TCA=>TTA	S=>L	0.8
ndhD	1423	475	CTT=>TTT	L=>F	0.8
ndhF	205	69	CAT=>TAT	H=>Y	0.8
ndhF	290	97	TCA=>TTA	S=>L	1
ndhF	586	196	CTT=>TTT	L=>F	0.8
ndhG	166	56	CAT=>TAT	H=>Y	0.8
ndhG	314	105	ACA=>ATA	T=>I	0.8
psaI	44	15	GCA=>GTA	A=>V	1
psbE	214	72	CCT=>TCT	P=>S	1
psbF	77	26	TCT=>TTT	S=>F	1
rpoA	893	298	TCA=>TTA	S=>L	1
rpoB	338	113	TCT=>TTT	S=>F	1
rpoB	551	184	TCA=>TTA	S=>L	1

rpoB	566	189	TCG=>TTG	S=>L	1
rpoB	2432	811	TCA=>TTA	S=>L	0.86
rpoC1	41	14	TCA=>TTA	S=>L	1
rpoC1	1717	573	CAC=>TAC	H=>Y	1
rpoC1	1946	649	ACT=>ATT	T=>I	0.86
rpoC2	2287	763	CGG=>TGG	R=>W	1
rpoC2	2336	779	GCC=>GTC	A=>V	0.86
rps2	248	83	TCA=>TTA	S=>L	1
rps14	80	27	TCA=>TTA	S=>L	1
rps14	149	50	TCA=>TTA	S=>L	1
rps16	176	59	TCA=>TTA	S=>L	0.83

Table S6. Predicted RNA editing site in the *Cadaba glandulosa* chloroplast genome

Gene	Nucleotide Position	Amino Acid Position	Codon	Amino Acid	Score
accD	800	267	TCG=>TTG	S=>L	0.8
atpF	92	31	CCA=>CTA	P=>L	0.86
clpP	562	188	CAT=>TAT	H=>Y	1
matK	706	236	CAT=>TAT	H=>Y	1
matK	1250	417	TCA=>TTA	S=>L	0.86
matK	1309	437	CAC=>TAC	H=>Y	1
ndhA	8	3	ACT=>ATT	T=>I	1
ndhA	341	114	TCA=>TTA	S=>L	1
ndhB	149	50	TCA=>TTA	S=>L	1
ndhB	467	156	CCA=>CTA	P=>L	1
ndhB	586	196	CAT=>TAT	H=>Y	1
ndhB	611	204	TCA=>TTA	S=>L	0.8
ndhB	746	249	TCT=>TTT	S=>F	1
ndhB	830	277	TCA=>TTA	S=>L	1
ndhB	836	279	TCA=>TTA	S=>L	1
ndhB	1255	419	CAT=>TAT	H=>Y	1
ndhB	1481	494	CCA=>CTA	P=>L	1
ndhD	20	7	ACG=>ATG	T=>M	1
ndhD	65	22	TCT=>TTT	S=>F	0.8
ndhD	401	134	TCA=>TTA	S=>L	1
ndhD	692	231	TCA=>TTA	S=>L	1
ndhD	896	299	TCA=>TTA	S=>L	1
ndhD	905	302	CCT=>CTT	P=>L	1
ndhD	1328	443	TCA=>TTA	S=>L	0.8
ndhD	1423	475	CTT=>TTT	L=>F	0.8
ndhF	205	69	CAT=>TAT	H=>Y	0.8
ndhF	290	97	TCA=>TTA	S=>L	1

ndhF	586	196	CTT=>TTT	L=>F	0.8
ndhF	2002	668	CAC=>TAC	H=>Y	1
ndhG	166	56	CAT=>TAT	H=>Y	0.8
ndhG	314	105	ACA=>ATA	T=>I	0.8
psaI	44	15	GCA=>GTA	A=>V	1
psbE	214	72	CCT=>TCT	P=>S	1
psbF	77	26	TCT=>TTT	S=>F	1
rpoA	887	296	TCA=>TTA	S=>L	1
rpoB	338	113	TCT=>TTT	S=>F	1
rpoB	551	184	TCA=>TTA	S=>L	1
rpoB	566	189	TCG=>TTG	S=>L	1
rpoB	2432	811	TCA=>TTA	S=>L	0.86
rpoC1	41	14	TCA=>TTA	S=>L	1
rpoC1	1717	573	CAC=>TAC	H=>Y	1
rpoC1	1946	649	ACT=>ATT	T=>I	0.86
rpoC2	2293	765	CGG=>TGG	R=>W	1
rpoC2	2342	781	GCC=>GTC	A=>V	0.86
rps2	248	83	TCA=>TTA	S=>L	1
rps14	80	27	TCA=>TTA	S=>L	1
rps14	149	50	TCA=>TTA	S=>L	1
rps16	176	59	TCA=>TTA	S=>L	0.83

Table S7. Predicted RNA editing site in the*Maerua crassifolia*chloroplast genome

Gene	Nucleotide Position	Amino Acid Position	Codon	Amino Acid	Score
accD	797	266	TCG=>TTG	S=>L	0.8
atpF	98	33	CCA=>CTA	P=>L	0.86
clpP	562	188	CAT=>TAT	H=>Y	1
matK	706	236	CAT=>TAT	H=>Y	1
matK	1250	417	TCA=>TTA	S=>L	0.86
matK	1309	437	CAC=>TAC	H=>Y	1
ndhA	8	3	ACT=>ATT	T=>I	1
ndhA	341	114	TCA=>TTA	S=>L	1
ndhB	149	50	TCA=>TTA	S=>L	1
ndhB	467	156	CCA=>CTA	P=>L	1
ndhB	586	196	CAT=>TAT	H=>Y	1
ndhB	611	204	TCA=>TTA	S=>L	0.8
ndhB	746	249	TCT=>TTT	S=>F	1

ndhB	830	277	TCA=>TTA	S=>L	1
ndhB	836	279	TCA=>TTA	S=>L	1
ndhB	1255	419	CAT=>TAT	H=>Y	1
ndhB	1481	494	CCA=>CTA	P=>L	1
ndhD	20	7	ACG=>ATG	T=>M	1
ndhD	65	22	TCT=>TTT	S=>F	0.8
ndhD	401	134	TCA=>TTA	S=>L	1
ndhD	692	231	TCA=>TTA	S=>L	1
ndhD	896	299	TCA=>TTA	S=>L	1
ndhD	905	302	CCT=>CTT	P=>L	1
ndhD	1328	443	TCA=>TTA	S=>L	0.8
ndhD	1423	475	CTT=>TTT	L=>F	0.8
ndhF	205	69	CAT=>TAT	H=>Y	0.8
ndhF	290	97	TCA=>TTA	S=>L	1
ndhF	586	196	CTT=>TTT	L=>F	0.8
ndhF	2002	668	CAC=>TAC	H=>Y	1
ndhG	166	56	CAT=>TAT	H=>Y	0.8
ndhG	314	105	ACA=>ATA	T=>I	0.8
psaI	44	15	GCA=>GTA	A=>V	1
psbE	214	72	CCT=>TCT	P=>S	1
psbF	77	26	TCT=>TTT	S=>F	1
rpoA	887	296	TCA=>TTA	S=>L	1
rpoB	338	113	TCT=>TTT	S=>F	1
rpoB	551	184	TCA=>TTA	S=>L	1
rpoB	566	189	TCG=>TTG	S=>L	1
rpoB	2432	811	TCA=>TTA	S=>L	0.86
rpoC1	41	14	TCA=>TTA	S=>L	1
rpoC1	1717	573	CAC=>TAC	H=>Y	1
rpoC1	1946	649	ACT=>ATT	T=>I	0.86
rpoC2	2293	765	CGG=>TGG	R=>W	1
rpoC2	2342	781	GCC=>GTC	A=>V	0.86
rps2	248	83	TCA=>TTA	S=>L	1
rps14	80	27	TCA=>TTA	S=>L	1
rps14	149	50	TCA=>TTA	S=>L	1
rps16	176	59	TCA=>TTA	S=>L	0.83

Table S8. Predicted RNA editing site in the *Maerua oblongifolia* chloroplast genome

Gene	Nucleotide Position	Amino Acid Position	Codon	Amino Acid	Score
accD	797	266	TCG=>TTG	S=>L	0.8
atpF	92	31	CCA=>CTA	P=>L	0.86
clpP	562	188	CAT=>TAT	H=>Y	1

matK	706	236	CAT=>TAT	H=>Y	1
matK	1250	417	TCA=>TTA	S=>L	0.86
matK	1309	437	CAC=>TAC	H=>Y	1
ndhA	8	3	ACT=>ATT	T=>I	1
ndhA	125	42	ACA=>ATA	T=>I	0.8
ndhA	341	114	TCA=>TTA	S=>L	1
ndhB	155	52	TCA=>TTA	S=>L	1
ndhB	473	158	CCA=>CTA	P=>L	1
ndhB	592	198	CAT=>TAT	H=>Y	1
ndhB	617	206	TCA=>TTA	S=>L	0.8
ndhB	752	251	TCT=>TTT	S=>F	1
ndhB	836	279	TCA=>TTA	S=>L	1
ndhB	842	281	TCA=>TTA	S=>L	1
ndhB	1261	421	CAT=>TAT	H=>Y	1
ndhB	1487	496	CCA=>CTA	P=>L	1
ndhD	20	7	ACG=>ATG	T=>M	1
ndhD	65	22	TCT=>TTT	S=>F	0.8
ndhD	401	134	TCA=>TTA	S=>L	1
ndhD	692	231	TCA=>TTA	S=>L	1
ndhD	896	299	TCA=>TTA	S=>L	1
ndhD	905	302	CCT=>CTT	P=>L	1
ndhD	1316	439	TCA=>TTA	S=>L	0.8
ndhD	1328	443	TCA=>TTA	S=>L	0.8
ndhD	1423	475	CTT=>TTT	L=>F	0.8
ndhF	205	69	CAT=>TAT	H=>Y	0.8
ndhF	290	97	TCA=>TTA	S=>L	1
ndhF	586	196	CTT=>TTT	L=>F	0.8
ndhG	166	56	CAT=>TAT	H=>Y	0.8
ndhG	314	105	ACA=>ATA	T=>I	0.8
psaI	44	15	GCA=>GTA	A=>V	1
psbF	77	26	TCT=>TTT	S=>F	1
rpl20	26	9	ACA=>ATA	T=>I	0.86
rpoA	887	296	TCA=>TTA	S=>L	1
rpoB	338	113	TCT=>TTT	S=>F	1
rpoB	551	184	TCA=>TTA	S=>L	1
rpoB	566	189	TCG=>TTG	S=>L	1
rpoB	2432	811	TCA=>TTA	S=>L	0.86
rpoC1	41	14	TCA=>TTA	S=>L	1
rpoC1	1717	573	CAC=>TAC	H=>Y	1
rpoC1	1946	649	ACT=>ATT	T=>I	0.86
rpoC2	2287	763	CGG=>TGG	R=>W	1
rpoC2	2336	779	GCC=>GTC	A=>V	0.86
rps2	248	83	TCA=>TTA	S=>L	1

rps14	149	50	TCA=>TTA	S=>L	1
rps16	176	59	TCA=>TTA	S=>L	0.83

Table S9. Repeat sequences present in the *Cadaba farinose* chloroplast genome

SN	Repeat Size	Repeat Position 1	Repeat Type	Repeat Location	Repeat Position 2	Repeat Location 2	E-Value
1	64	39696	F	*psaB*	41920	*psaA*	2.02E-29
2	44	75442	P	IGS	75442	IGS	2.23E-17
3	34	29585	P	IGS	29585	IGS	2.33E-11
4	30	8202	P	IGS	45811	*trnS-GGA*	5.97E-09
5	28	127142	P	IGS	127142	IGS	9.56E-08
6	26	9740	P	IGS	9772	IGS	1.53E-06
7	26	61740	P	*cemA*	61740	*cemA*	1.53E+00
8	24	31569	P	IGS	31569	IGS	2.45E-05
9	24	115382	P	IGS	115382	IGS	2.45E-05
10	23	114633	R	IGS	114633	IGS	9.79E-05
11	22	5279	R	IGS	5279	IGS	3.91E-04
12	22	90517	F	*ycf2*	90538	*ycf2*	3.91E-04
13	22	90517	P	*ycf2*	151486	*ycf2*	3.91E-04
14	22	90538	P	*ycf2*	151507	*ycf2*	3.91E-04
15	22	111580	P	*ycf1*	111580	*ycf1*	3.91E-04
16	22	111580	F	*ycf1*	130444	IGS	3.91E-04
17	22	130444	P	IGS	130444	IGS	3.91E-04
18	22	151486	F	*ycf2*	151507	*ycf2*	3.91E-04
19	21	8208	F	*trnS-GCU*	36412	*trnS-UGA*	1.57E-03
20	21	30738	P	IGS	72595	*clpP* Intron	1.57E-03
21	21	36412	P	*trnS-UGA*	45814	*trnS-GGA*	1.57E-03
22	21	37671	F	*trnfM-CAU*	67641	*trnP-UGG* *trnP-GGG*	1.57E-03
23	21	90484	F	*ycf2*	90529	*ycf2*	1.57E-03
24	21	90484	P	*ycf2*	151496	*ycf2*	1.57E-03
25	21	90529	P	*ycf2*	151541	*ycf2*	1.57E-03
26	21	114445	P	IGS	114913	IGS	1.57E-03
27	21	114538	R	IGS	114538	IGS	1.57E-03
28	21	151496	F	*ycf2*	151541	*ycf2*	1.57E-03
29	20	370	P	IGS	370	IGS	6.26E-03
30	20	3909	P	IGS	3909	IGS	6.26E-03
31	20	4886	P	IGS	4886	IGS	6.26E-03
32	20	13855	F	IGS	89473	*ycf2*	6.26E-03
33	20	13855	P	IGS	152553	*ycf2*	6.26E-03
34	20	28661	F	IGS	55576	IGS	6.26E-03

35	20	36480	P	*trnS-UGA*	45753	IGS	6.26E-03
36	20	44401	R	IGS	65664	IGS	6.26E-03
37	20	48417	P	*trnL-UAA* Intron	66939	IGS	6.26E-03
38	20	51127	R	*ndhC*	51127	*ndhC*	6.26E-03
39	19	325	P	IGS	5279	IGS	2.51E-02
40	19	325	C	IGS	5282	IGS	2.51E-02
41	19	4650	R	IGS	4650	IGS	2.51E-02
42	19	4760	C	IGS	114444	IGS	2.51E-02
43	19	4761	C	IGS	61352	IGS	2.51E-02
44	19	4763	R	IGS	4763	IGS	2.51E-02
45	19	8719	R	IGS	8719	IGS	2.51E-02
46	19	9654	F	IGS	37505	*trnG-UCC*	2.51E-02
47	19	15183	R	*atpI*	15183	*atpI*	2.51E-02
48	19	23265	P	*rpoC1* Intron	114912	IGS	2.51E-02
49	19	23267	F	*rpoC1* Intron	53212	IGS	2.51E-02

Table S10. Repeat sequences present in the *Cadaba glandulosa* chloroplast genome

SN	Repeat Size	Repeat Position 1	Repeat Type	Repeat Location	Repeat Position 2	Repeat Location 2	E-Value
1	64	39682	F	*psaB*	41906	*psaA*	2.03E-29
2	44	75578	P	IGS	75578	IGS	2.23E-17
3	39	0	P	IGS	85642	*rps19*	2.28E-14
4	36	29576	P	IGS	29576	IGS	1.46E-12
5	30	8188	P	IGS	45811	*trnS-GGA*	5.98E-09
6	28	67079	P	IGS	67079	IGS	9.57E-08
7	28	127227	P	IGS	127227	IGS	9.57E-08
8	26	9774	P	IGS	9806	IGS	1.53E-06
9	26	10148	P	IGS	10148	IGS	1.53E-06
10	26	61883	P	*cemA*	61883	*cemA*	1.53E-06
11	24	27878	P	IGS	27878	IGS	2.45E-05
12	24	31593	P	IGS	31593	IGS	2.45E-05
13	24	115478	P	IGS	115478	IGS	2.45E-05
14	22	51600	R	IGS	51600	IGS	3.92E-04
15	22	90595	F	*ycf2*	90616	*ycf2*	3.92E-04
16	22	90595	P	*ycf2*	151603	*ycf2*	3.92E-04
17	22	90616	P	*ycf2*	151624	*ycf2*	3.92E-04
18	22	111681	P	*ycf1*	111681	*ycf1*	3.92E-04
19	22	111681	F	*ycf1*	130538	IGS	3.92E-04
20	22	130538	P	IGS	130538	IGS	3.92E-04
21	22	151603	F	*ycf2*	151624	*ycf2*	3.92E-04

22	21	8194	F	*trnS-GCU*	36410	*trnS-UGA*	1.57E-03
23	21	8406	R	IGS	8406	IGS	1.57E-03
24	21	10188	P	IGS	10214	IGS	1.57E-03
25	21	36410	P	*trnS-UGA*	45814	*trnS-GGA*	1.57E-03
26	21	37674	F	*trnfM-CAU*	67774	*trnP-UGG trnP-GGG*	1.57E-03
27	21	48431	P	*trnL-UAA* Intron	67082	IGS	1.57E-03
28	21	48431	F	*trnL-UAA* Intron	67083	IGS	1.57E-03
29	21	49478	C	IGS	109765	IGS	1.57E-03
30	21	49478	R	IGS	132455	IGS	1.57E-03
31	21	61505	F	IGS	114548	IGS	1.57E-03
32	21	71728	R	*clpP* Intron	71728	*clpP* Intron	1.57E-03
33	21	90562	F	*ycf2*	90607	*ycf2*	1.57E-03
34	21	90562	P	*ycf2*	151613	*ycf2*	1.57E-03
35	21	90607	P	*ycf2*	151658	*ycf2*	1.57E-03
36	21	151613	F	*ycf2*	151658	*ycf2*	1.57E-03
37	20	281	R	IGS	109767	IGS	6.27E-03
38	20	281	C	IGS	132454	IGS	6.27E-03
39	20	282	R	IGS	282	IGS	6.27E-03
40	20	289	C	IGS	114554	IGS	6.27E-03
41	20	4854	P	IGS	4854	IGS	6.27E-03
42	20	13886	F	IGS	89551	*ycf2*	6.27E-03
43	20	13886	P	IGS	152670	*ycf2*	6.27E-03
44	20	36478	P	*trnS-UGA*	45753	IGS	6.27E-03
45	20	44406	R	IGS	65814	IGS	6.27E-03
46	20	48431	P	*trnL-UAA* Intron	48431	*trnL-UAA* Intron	6.27E-03
47	20	51151	R	*ndhC*	51151	*ndhC*	6.27E-03
48	20	51622	F	IGS	51635	IGS	6.27E-03
49	19	282	P	IGS	49480	IGS	2.51E-02

Table S11. Repeat sequences present in the *Maerua crassifolia* chloroplast genome

SN	Repeat Size	Repeat Position 1	Repeat Type	Repeat Location	Repeat Position 2	Repeat Location 2	E-Value
1	52	0	P	IGS	84572	*rps19*	3.36E-22
2	52	29460	P	IGS	29460	IGS	3.36E-22
3	52	39525	F	*psaB*	41749	*psaA*	3.36E-22
4	43	33135	F	IGS	33156	IGS	8.81E-17
5	42	74663	P	IGS	74663	IGS	3.52E-16
6	30	8240	P	IGS	45697	IGS	5.91E-09

7	25	116737	P	IGS	116813	IGS	6.05E-06
8	24	31484	P	IGS	31484	IGS	2.42E-05
9	24	124714	P	IGS	124714	IGS	2.42E-05
10	22	418	P	IGS	418	IGS	3.87E-04
11	22	33135	F	IGS	33177	IGS	3.87E-04
12	22	89527	F	*ycf2*	89548	*ycf2*	3.87E-04
13	22	89527	P	*ycf2*	150739	*ycf2*	3.87E-04
14	22	89548	P	*ycf2*	150760	*ycf2*	3.87E-04
15	22	110500	P	*ycf1*	110500	*ycf1*	3.87E-04
16	22	110500	F	*ycf1*	129787	IGS	3.87E-04
17	22	129787	P	IGS	129787	IGS	3.87E-04
18	22	150739	F	ycf2	150760	ycf2	3.87E-04
19	21	8246	F	*trnS-GCU*	36291	*trnS-UGA*	1.55E-03
20	21	10044	R	IGS	10044	IGS	1.55E-03
21	21	36291	p	*trnS-UGA*	45700	*trnS-UGA*	1.55E-03
22	21	37504	F	*trnfM-CAU*	66941	*trnP-UGG trnP-GGG*	1.55E-03
23	21	113435	R	IGS	113435	IGS	1.55E-03
24	21	114388	P	IGS	114450	IGS	1.55E-03
25	20	13709	F	IGS	88483	*ycf2*	6.20E-03
26	20	13709	P	IGS	151806	*ycf2*	6.20E-03
27	20	36359	P	*trnS-UGA*	45639	IGS	6.20E-03
28	20	44299	R	IGS	65236	IGS	6.20E-03
29	20	48298	F	IGS	66266	IGS	6.20E-03
30	20	63710	P	IGS	82317	IGS	6.20E-03
31	19	8763	R	IGS	113809	IGS	2.48E-02
32	19	9729	F	IGS	37333	*trnG-UCC*	2.48E-02
33	19	10044	F	IGS	63644	IGS	2.48E-02
34	19	10046	R	IGS	63644	IGS	2.48E-02
35	19	15102	R	*atpI*	15102	*atpI*	2.48E-02
36	19	37154	R	IGS	37154	IGS	2.48E-02
37	19	42892	R	IGS	42892	IGS	2.48E-02
38	19	47004	R	IGS	47004	IGS	2.48E-02
39	19	48900	F	*trnF-GAA*	104247	*atpA*	2.48E-02
40	19	48900	P	*trnF-GAA*	136043	*trnA-UGC* Exon	2.48E-02
41	19	62026	R	*trnF-GAA*	62026	*trnF-GAA*	2.48E-02
42	19	64086	F	*trnF-GAA*	121855	*ndhA* Intron	2.48E-02
43	19	78489	R	*rpoA*	78489	*rpoA*	2.48E-02
44	19	94433	R	IGS	94433	IGS	2.48E-02
45	19	94433	C	IGS	145857	IGS	2.48E-02
46	19	109015	R	IGS	128188	IGS	2.48E-02

47	19	113340	R	IGS	113340	IGS	2.48E-02
48	19	114412	R	IGS	114412	IGS	2.48E-02
49	19	115515	R	IGS	115515	IGS	2.48E-02

Table S12. Repeat sequences present in the *Maerua oblongifolia* chloroplast genome

SN	Repeat Size	Repeat Position 1	Repeat Type	Repeat Location	Repeat Position 2	Repeat Location 2	E-Value
1	52	29639	P	IGS	29639	IGS	3.35E-22
2	52	39179	F	*psaB*	41403	*psaA*	3.35E-22
3	42	74217	P	IGS	74217	IGS	3.51E-16
4	30	8263	P	IGS	45323	IGS	5.89E-09
5	28	126072	P	IGS	126072	IGS	9.43E-08
6	26	388	P	IGS	423	IGS	1.51E-06
7	26	9800	P	IGS	9832	IGS	1.51E-06
8	26	10040	P	IGS	10040	IGS	1.51E-06
9	26	31157	P	IGS	31157	IGS	1.51E-06
10	23	0	P	IGS	84130	*rps19*	9.66E-05
11	22	70135	P	*clpP* Intron	100101	*rrn16*	3.86E-04
12	22	70135	F	*clpP* Intron	139466	IGS	3.86E-04
13	22	89084	F	*ycf2*	89105	*ycf2*	3.86E-04
14	22	89084	P	*ycf2*	150462	*ycf2*	3.86E-04
15	22	89105	P	*ycf2*	150483	*ycf2*	3.86E-04
16	22	110133	P	*ycf1*	110133	*ycf1*	3.86E-04
17	22	110133	F	*ycf1*	129434	IGS	3.86E-04
18	22	129434	P	IGS	129434	IGS	3.86E-04
19	22	150462	F	*ycf2*	150483	*ycf2*	3.86E-04
20	21	8269	F	*trnS-GCU*	35920	*trnS-UGA*	1.55E-03
21	21	10033	R	IGS	10033	IGS	1.55E-03
22	21	35920	P	*trnS-UGA*	45326	IGS	1.55E-03
23	21	37155	F	*trnfM-CAU*	66481	*trnP-UGG* *trnP-GGG*	1.55E-03
24	21	50040	R	*ndhK- psbG*	50040	*ndhK- psbG*	1.55E-03
25	21	113074	R	IGS	113074	IGS	1.55E-03
26	21	114033	P	IGS	114095	IGS	1.55E-03
27	21	114428	R	IGS	114428	IGS	1.55E-03
28	21	116403	P	IGS	116428	IGS	1.55E-03
29	20	5170	P	IGS	5194	IGS	6.18E-03
30	20	8398	P	IGS	54240	*atpB*	6.18E-03
31	20	8707	P	IGS	116404	IGS	6.18E-03
32	20	8707	F	IGS	116428	IGS	6.18E-03
33	20	35988	P	*trnS-UGA*	45265	IGS	6.18E-03

34	20	47912	F	IGS	65793	IGS	6.18E-03
35	20	50234	R	*ndhC*	50234	*ndhC*	6.18E-03
36	20	75118	R	IGS	75118	IGS	6.18E-03
37	19	1742	P	IGS	59660	IGS	2.47E-02
38	19	6921	P	IGS	6942	IGS	2.47E-02
39	19	9714	F	IGS	36982	*trnG-UCC*	2.47E-02
40	19	13903	F	*atpH*	36234	IGS	2.47E-02
41	19	15301	R	*atpI*	15301	*atpI*	2.47E-02
42	19	32992	C	IGS	82412	IGS	2.47E-02
43	19	46463	F	IGS	50040	*ndhK- psbG*	2.47E-02
44	19	46463	R	IGS	50042	*ndhK -psbG*	2.47E-02
45	19	48513	F	*trnF-GAA*	103817	*trnA-UGC* Exon	2.47E-02
46	19	48513	P	*trnF-GAA*	135753	*trnA-UGC* Exon	2.47E-02
47	19	50048	R	*ndhK- psbG*	50048	*ndhK psbG*	2.47E-02
48	19	63240	R	IGS	78046	*rpoA*	2.47E-02
49	19	78044	R	*rpoA*	78044	*rpoA*	2.47E-02
